# Pain after 1940 nm Laser for Unilateral Incompetence of the Great Saphenous Vein

**DOI:** 10.3390/jcm13133839

**Published:** 2024-06-29

**Authors:** Torsten Willenberg, Simon Bossart, Michael Schubert, Sarvesh Ghorpade, Axel Haine

**Affiliations:** 1 Gefässzentrum Bern, VASC Angiologie und Intervention, Lindenhofspital Bern, 3012 Bern, Switzerland; axel.haine@gefaesszentrum-bern.ch; 2 Department of Dermatology, Inselspital, Bern University Hospital, University of Bern, 3010 Bern, Switzerland; simon.bossart@insel.ch; 3 iMS GmbH, 82327 Tutzing, Germany; michael.schubert@ims-medical.de (M.S.); sarvesh.ghorpade@ims-medical.de (S.G.)

**Keywords:** varicose veins, laser, pain

## Abstract

**Background:** To investigate postprocedural pain after using an endovenous 1940 nm laser for great saphenous vein incompetence. **Methods:** A total of 72 patients were treated for symptomatic unilateral great saphenous incompetence using a 1940 nm laser device. All patients were treated using a standardized procedure under local anesthesia and investigated for postprocedural pain for 4 weeks using a visual analog scale (VAS 0-10). **Results:** Moderate pain was reported. A total of 17 patients reported minor scale 1 after the first day. On average, pain regressed to minor 1 after day 6. No significant complications were observed. **Conclusions:** Our results support the atraumatic character of this higher wavelength laser. In terms of patient comfort, higher wave lengths such as 1940 nm should be preferred for endovenous laser ablation. Using a combination of wavelengths could be the future solution to providing both safe ablation and minimum postprocedural pain.

## 1. Introduction

The endovenous laser technique for treating superficial venous reflux is today the golden standard procedure. Significant technical improvements have been made in this minimal invasive approach over the last two decades, surpassing other methods such as radiofrequency ablation or non-tumescence-based techniques [1].

Endovenous laser light emission has transitioned to a homogeneous radial pattern to achieve complete destruction of the entire venous wall while minimizing the risk of localized accumulation of laser energy, which could lead to perforations and associated local side effects such as pain and tissue damage. Wavelengths have advanced from 810 nm to 1940 nm, targeting different chromophores. The higher wavelengths (1320 nm, 1470 nm, and 1940 nm) target water and act specifically on the vessel wall. Therefore, they seem to be very effective in terms of occlusion rates and are reported to exceed 90% [2].

Previously, we investigated the course of post-interventional pain after 1470 nm for laser ablation of the great saphenous vein [3]. In this study, higher energy application was correlated with increased post-interventional pain within the first two weeks after treatment. We assumed that the higher wavelength of 1940 nm, requiring less energy per/cm^2^, might lead to reduced post-interventional pain. Accordingly, we then examined a cohort of patients treated with this wavelength for primary reflux of the great saphenous vein.

## 2. Methods

A total of 72 consecutive patients referred for minimal invasive treatment of unilateral, complete great saphenous vein (GSV) incompetence—confirmed by prior duplex sonographic evaluation based on a pathological reflux of >0.5 s in the GSV after distal manual compression of the calf—were asked to take part in the investigation. Patients were advised to fill out a VAS pain score (0–10, 0 = no pain, 10 = strongest pain) for 28 days from day 1, which was defined as the day of the intervention. The score was defined as representing the highest sensation of pain or discomfort in the treated leg during each of the 28 days.

Exclusion criteria were defined as the following:

Recurrent GSV incompetence, bilateral GSV treatment, any leg pain for other reasons than varicose veins, any persistent anti-inflammatory medication including NSAID, venous ulceration, a history of deep vein thrombosis.

The minimally invasive treatment procedure was standardized as follows: Endovenous laser for the insufficient GSV was applied using a 1940 nm device (iMS GmbH, Tutzing, Germany) 4 French introducer sheet) under tumescent anaesthesia (solution: 500 mL Saline, 20 mL Rapidocain^®^, Sintetica SA, Mendrisio, Switzerland). Laser power was fixed at 6 W, the laser fibre was withdrawn at a rate of approximately 0.1–0.2 cm/s. This rate was adapted according to the experience of the operators considering the diameter of the treated vein. In general, an energy application of 35–55 J/cm was considered to be efficient. The laser tip was placed right at the confluence of the epigastric vein or at least 0.5 cm distal from the sapheno-femoral junction. All procedures were carried out in an ambulatory setting under local anaesthesia without any sedation.

Side branches were treated with avulsions or with ultrasound guided foam sclerotherapy. The latter was performed using 1–3% polidocanol foam (liquid/air ratio 1:4). The choice of concentration was made based on the diameter of the treated vein: in general, using 1% foam, for larger veins (>1 cm diameter), using 3% foam.

All patients received a single injection of Enoxaparine Sodium (Clexane^®^, Sanofi, Le Trait, France) 40mg < 70 kg body weight, 60 mg > 70 kg body weight) at the end of the procedure and were asked to take Rivaroxaban 10 mg/d for a further 3 days. After the procedure, the treated leg was bandaged with a self-stitching bandage (Peha Haft^®^, Paul Hartmann AG, Heidenheim, Germany) and a Swiss class II thigh-high compression stocking (23–32 mmHg) was applied over this bandage. Patients were asked to maintain this compression system for two nights and carry on with the compression stocking during the daytime for another 14 days thereafter. After this period, compression was advised on demand. Painkillers were not suggested as a fixed regimen but were advised on demand.

Two follow up visits were carried out: the first one, within 7 days after treatment, the second, after 6 to 8 weeks.

Several parameters were defined for registration: body mass index as kg/m^2^, C-score from the CEAP-Classification (C0–C6), GSV diameter defined as the maximum diameter in standing position at 1–5 cm distal from the sapheno-femoral junction, applied energy per centimetre of the treated GSV (joule/cm) and length of the treated segment (VS).

To analyze the VAS pain score over a period of 28 days, the arithmetic mean (aver-age) for 72 patients from day 1 to day 28 was calculated. The arithmetic mean (average) per day was then compared to the level of VAS pain on a scale from 1 to 10. Spearman’s rank correlation was calculated for average VAS pain for 28 days for different types of parameters correlated to patient treatment. Microsoft^®^ Excel^®^ 2019 MSO (Version 2401 Build 16.0.17231.20236) 32 Bit was used for the calculation.

## 3. Results

The basic data of 72 patients are shown in Table 1.

**Figure 1:**
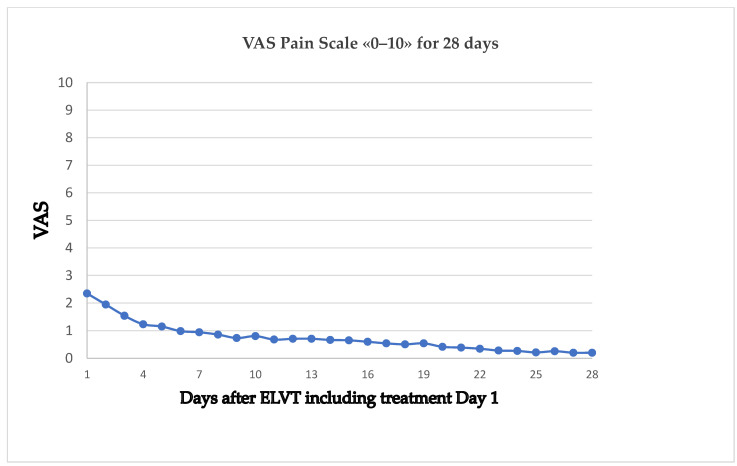
Pain scale average of 72 patients after 1940 nm laser treatment for 28 days.

The pain scale average for all patients is shown in Figure 1. Pain regressed continuously to an average scale of less than 1 at day 6.

**Figure 2:**
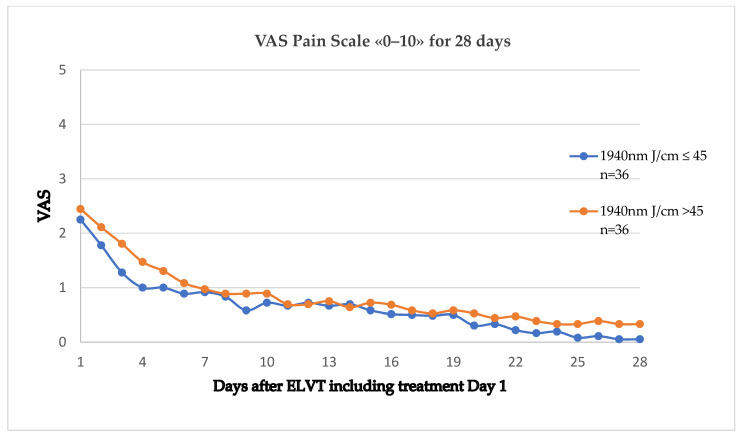
Pain scale average divided into 2 groups: n = 36 with linear endovenous energy density < 45 Joule/cm, yellow, n = 36 with linear endovenous energy density > 45 Joule/cm, blue.

The pain scale divided into energy application <45 Joule/cm and >45 Joule/cm is shown in Figure 2. A moderate tendency to less pain was noted in the lower energy group. The difference was not statistically significant (*p* = 0.97).

No difference was noted in pain scale between patients with a BMI > 30 kg/m^2^ and < 30 kg/m^2^ (*p* = 0.93)

No difference was noted in pain scale between patients with up to and with more than 20 cm of additional avulsions (*p* = 0.95).

The same was seen between patients with additional foam sclerotherapy and without additional foam sclerotherapy (*p* = 0.91).

No significant complications such as DVT or PE were observed in the treated cohort.

## 4. Discussion

In this series of patients treated for complete GSV incompetence using a 1940 nm endovenous laser device, we investigated postprocedural pain over 28 days. Our results show a low pain scale, which decreases on average to less than 1 out of 10 after 6 days. These findings support our hypothesis that endovenous laser treatment (ELVT) with a 1940 nm wavelength causes minimal trauma and therefore minimal postprocedural pain. This observation is in line with the physical activity of 1940 nm wavelength, whose chromophore specifically targets water molecules within the endothelial layer, limiting its effect to the venous wall without penetrating the surrounding tissue.

Our findings suggest that higher wavelengths should be preferred in terms of patient comfort following the procedure. However, based on our experience with different wavelength generators, lower wavelengths obviously provide more power and may be preferred for larger diameters or other circumstances requiring more energy—like, for example, a post-thrombotic state of the GSV—for safe and permanent occlusion of the treated insufficient vein. The choice of technical support by various laser devices has grown over the recent years. Thus, a combination of different techniques could be of interest to optimize the outcome in terms of both efficiency and comfort.

We also compared two different levels of energy application within our series, one with <45 Joule/cm and the other with ≥45 Joule/cm. A higher energy application can be achieved by a slower rate of laser fibre withdrawal. Moderately less pain was noted in the lower energy group. This tendency is in line with our previous study and our hypothesis that less energy causes less tissue trauma and inflammation [3].

An interesting option for modern laser treatment is the ability to change the wavelengths within the procedure using one and the same fibre. For instance, applying the lower wavelengths for the thigh region, where the diameter of the GSV is usually more important and switching to the higher wavelengths for the calf region may result in reduced pain and potentially less nerve irritation, one of the very few but constantly observed side effects after endovenous laser treatment. This side effect is due to the proximity of the saphenous nerve to the GSV, below the knee, near the vein [4]. We have already implemented this technique in an off-label setting using the same single fibre with a different wavelength generator, resulting in safe and successful procedures without technical or procedural issues.

This technical approach could represent another step towards the “optimal” tailored procedure by ensuring safe occlusion of the proximal trunk while preserving surrounding tissue at the distal parts of the GSV. However, this hypothesis requires further scientific validation.

Our study has several limitations. It is a single series without a control group. Direct comparison with another wavelength is not reliable. We thought to compare the results of this 1940 nm series to a previous published cohort treated with the 1470 nm device. However, the basic data of the two cohorts differed substantially like, for instance, the maximum vein diameter; thus, a comparison of the two groups would be scientifically incorrect. Moreover, our patients could be biased to report less pain as the operator was also providing the follow-up visits. Moreover, other non-observed factors like postprocedural physical activity, the use of pain killers and compliance to the compression treatment could have influenced the sensation of pain. However, regarding the additional treatment modalities like phlebectomy and sclerotherapy, which were applied for the side branches, we did not note any difference when the modality was applied more, less or not at all. Another limitation of our study is the potential presence of the Hawthorne effect. This effect means people might change their behavior because they know they are being watched. It can make patients report less pain to please the doctors.

In conclusion, our postprocedural pain report involving 72 patients treated for GSV incompetence with the highest available wavelength of 1940 nm supports the concept that this wavelength acts very specifically on the venous endothelium leading to reduced inflammation and pain in the aftermath of laser treatment for primary varicose veins. Further trials are necessary to determine the optimal combination of wavelength and energy and long-term occlusion rates over 5–10 years.

## Figures and Tables

**Table 1:** Basic data of 72 included patients.

Basic Data	1940 VSM		
		n	%
Age (years)	20–85	72	100
BMI kg/m^2^	≤30	65	90.27
	>30	7	9.72
Applied Energy per cm (Joule)	≤45	36	50
	>45	36	50
C-Stade CEAP Classification	2	20	27.78
	3	17	23.61
	4	33	45.83
	6	2	2.78
Diameter at sapheno-femoral junction (cm)	<1	61	84.72
	>1	11	15.27
Maximum Diameter (cm)	<1	57	79.16
	>1	15	20.83
Avulsions for sidebranches (cm)	≤20	36	50
	>20	36	50
Sklerotherapy for sidebranches	Yes	58	80.55
	No	14	19.45
Pain on Day 1	<1	17	23.62

## Data Availability

The raw data supporting the conclusions of this article will be made available by the authors on request.
